# *Entamoeba histolytica* and *Entamoeba dispar* infection in Mexican school children: genotyping and phylogenetic relationship

**DOI:** 10.1186/s12879-016-1812-8

**Published:** 2016-09-13

**Authors:** Liliana Rojas, Patricia Morán, Alicia Valadez, Alejandro Gómez, Enrique González, Eric Hernández, Oswaldo Partida, Miriam Nieves, Marco Gudiño, Ulises Magaña, Javier Torres, Cecilia Ximénez

**Affiliations:** 1 Research Unit of Experimental Medicine, Faculty of Medicine, National Autonomous University of Mexico (UNAM), Dr Balmis No148 Col. Doctores, CP 06726 Mexico City, Mexico; 2 Research Unit of infectious Diseases, Pediatric Hospital, XXI Century Medical Center, Mexican Institute of Social Security (IMSS), Mexico City, Mexico

**Keywords:** Frequency, E. histolytica, E. dispar, Genotype, Phylogenetic relationship, Patterns of transmission

## Abstract

**Background:**

This study aimed to determine the frequency of *Entamoeba histolytica* and *Entamoeba dispar* infection in school children in the community of Tlaltizapan, in order to understand the dynamics of infection within the school and family spheres of this population. Amoebiasis is an unsolved public health problem and an endemic disease in Mexico. The incidence rate varies depending on the state; the most affected states show the highest numbers of new cases of amoebiasis per year. Previously, we reported the molecular frequency of infection with *E. histolytica* and/or *E. dispar* in other rural communities of the state of Morelos.

**Methods:**

Children from 3 schools were studied to estimate the frequency of intestinal parasites through microscopic examination of fresh stool samples. The number of studied individuals were 309 school children. The molecular characterization of *E. histolytica* or *E. dispar* was carried out by Polymerase Chain Reaction (PCR) using species-specific primers to amplify short tandem repeats (STR) in non-coding sequences associated with the tRNA gene; the amplified fragments were sequenced and analyzed.

**Results:**

Eight different genotypes were obtained from *E. dispar* isolates with the molecular marker NKD3-D5. None of the cases in which the species *E. histolytica* was detected developed symptoms attributable to an invasive process of disease. Moreover, the parasitized condition appeared to have no significant impact on the development or nutritional status of affected children. Genotype 1, which corresponds to the reference strain *E. dispar* SAW760, considered a non-pathogenic amoeba, was the most prevalent.

**Conclusions:**

The comparison of the genotypes of *Entamoeba* species did not show a correlation between children and their relatives. In this community, the species *Entamoeba dispar* genotype 1 was the most widespread. Based on the indicators of growth, development and nutrition status, the studied community seems to be reasonably adapted to constant exposure to intestinal parasites, since there were no evidences of a serious impact of the parasitized condition on the children’s health.

## Background

Mexico has a highly diverse geography, and its sociodemographic conditions are even more diverse. This diversity is undoubtedly reflected on the morbidity rates of amoebiasis observed in different geographic areas of our country. The incidence rates of amoebiasis are different between the northern, northwestern, southeastern and highland regions of the country. However, in the last two decades, the age range of the population at risk has not changed; it still corresponds to children aged less than 18 years old. The highest rates are found in children aged 1–4 years (1034.04 cases/100,000 inhabitants in 2012) (the official incidence rate was 796.39 cases/100,000 inhabitants) [1].

Amoebiasis is one of the 20 main causes of disease in Mexico; however, the current frequency of infection due to both *E. histolytica* and *E. dispar* in the Mexican population is unknown. Some isolated epidemiological studies have been made using molecular tools to characterize *E. histolytica* and *E. dispar* [2–4].

Recently, we performed a trial study in an open population in the state of Morelos, and found a total prevalence of 21 % for infection with both species of *Entamoeba*, *E. histolytica* has a higher (1 3.8 %) compared with *E. dispar* (9.6 %) and mixed infections were 2.4 % [5]. Determining the real numbers of amoebic infection has been the aim of several studies in other endemic areas and in specific groups of individuals sharing close environments or with risky sexual practices; these studies have produced highly variable data [5–9]. Therefore, a worldwide estimation of the burden of disease due to each of the two species of amoebas [10] cannot currently be done.

Nevertheless, epidemiological studies of amoebiasis using molecular strategies have unveiled the extraordinary complexity of both species of *Entamoeba* and the genetic intra-specific variability in coding and in non-coding regions of DNA [5, 11–14]. Both species of *Entamoeba* are highly polymorphic; however, *E. histolytica* is clearly less polymorphic than *E. dispar* [14]. Now we know that both species have a peculiar geographic distribution [14], indicating that some genotypes have higher or lower geographic mobility; this characteristic can help us understand the patterns of transmission in specific communities or groups of individuals exposed to infection. Migrating infected individuals can become sources of new infections and potential outbreaks of amoebiasis, making the molecular tracking of *Entamoeba* isolates a valuable tool for global epidemiological and genealogical studies of *E. histolytica* and *E. dispar* [15]. The main objectives of the present work were to determine the molecular frequency and the dynamic of infection of *E. histolytica* and/or *E. dispar* in a school children population in Tlaltizapan, Morelos. For this purpose, we performed the genotyping of *Entamoeba* isolates and the phylogenetic reconstruction of parasite DNA sequences in order to detect the possible source of infection and the patterns of transmission. To estimate the impact of infection with parasitized condition on the studied children, we applied an index of anemia to both parasitized and non-parasitized children, and measured their body mass index according to the gender of the children.

## Methods

Study area and sampling. A cross-sectional study was conducted from January to May 2011 among 309 children from 3 different schools randomly selected in the village of Tlaltizapan, in the state of Morelos.

The sample size was calculated taking into account the total number of school children in the town (5,921) within the same range of age (5–14). The required size of the sample was calculated to be 190 children; the expected frequency of intestinal parasitic infection was 15 %; the worst acceptable level was 10 %; the confidence level was 95 %, and the results were considered statistical significant when *p* <0.05 % (Epi Info version 6) [16]. Even though the estimated sample size was less than the 309 individuals actually studied, the larger sample resulted in a strong statistical power, particularly for the frequency of the least prevalent parasites species.

The type of housing varies significantly in Tlaltizapan; in the center of the town there are well-constructed houses characteristic of urban settlements, with running water and drainage, electricity, and cement floors. However, the typical rural houses have no running water and no sewage facilities. In some cases, there is no latrine and the floors consist of compacted soil. The average number of inhabitants per house is seven individuals.

### Ethical considerations

The study protocol was submitted for evaluation and was approved by the Health Ministry of the state of Morelos; it was also approved by the ethical committee of the Faculty of Medicine of the National Autonomous University of Mexico (UNAM). In both cases, the ethical committees applied the Mexican Official Norm NOM-012-SSA3-2007, which deals with human and animal research, to support their decision to approve this research.

### Studied population

Three different schools were included in the study, all with similar characteristics and facilities (running water, toilet facilities, and 30–40 children per classroom). The age range studied was between 5 and 14 years, and there were no gender restrictions. The parents or legal guardians of each child, as well as the directors and teaching staff of the schools were informed about the details of the project, the advantages of voluntary participation, the sampling procedures and the potential risk of sampling. After the parents expressed their willingness to include their children in the study, they were asked to sign a letter of informed consent.

Each parent or guardian was interviewed by a member of our fieldwork team to assess the conditions of housing, access to potable water, waste disposal, and hygienic habits of the family. Pathological antecedents were also investigated, particularly recent episodes of diarrhea (in the previous 6 months). Information was collected through a previously validated questionnaire [5] and uploaded to a database for statistical analysis. Afterwards, wide mouth screw-capped containers, previously labeled with the complete name of the child and the respective code, were distributed to the parents or guardians with instructions for collecting and preserving the stool samples at home until they were collected by our fieldwork team.

With the purpose of determining the dynamics of transmission between each child and their near relatives, we also collected stool samples from the relatives of children who were microscopically positive for fecal parasites. A total of 167 stool samples were obtained from the relatives. The only condition to be included in the study was that the parasitized children and their relatives shared the same home.

### Microscopic detection of intestinal parasites

The stool samples were kept at 4 °C and transported to the Health Center in Tlaltizapan, and then to the laboratory in Mexico City. They were analyzed as previously reported [5]. Briefly, the fecal samples were suspended in 4 % Lugol’s iodine solution, and microscopic observation was performed at 10× and 40× magnification. To determine the concentration of ova and cysts, we used the Faust-Ferreira technique in the presence of a zinc-sulfate gradient solution (*d* = 1.192); a sample was collected from the flotation disk using a Pasteur pipette, suspended in 4 % Lugol’s solution and microscopically observed as described above [17].

### DNA extraction from stool samples

DNA was extracted from cysts found in stool samples subjected to the zinc-sulfate gradient flotation technique. Cysts were transferred to a 2 mL Eppendorf tube, washed 4 times with 0.15 M NaCl, and resuspended in 300 μl of lysis buffer (100 mM EDTA, pH 8, 0.25 % SDS) [18]. The tubes were subjected to five freezing cycles in ethanol-dry ice and thawed in a 37 °C water bath. Afterwards, 3 μl of 20 mg/mL of proteinase K were added. The samples were incubated for 1 h at 55 °C. After digestion with proteinase K, the lysates were brought to 0.7 M NaCl and 1 % CTAB (Sigma Chemical Co., St. Louis, MO). The mixture was incubated at 65 °C for 30 min and the samples were then extracted with chloroform, phenol/chloroform and chloroform, followed by precipitation of DNA with ethanol. DNA was suspended in 50 μl of water and passed over a Sephadex G-25 spin column (Pharmacia Biotech, Uppsala, Sweden).

### Molecular characterization of *E. histolytica* and *E. dispar*

The DNA obtained was used for amplification by polymerase chain reaction (PCR) in a 20 μl reaction mixture. Transfer RNA (tRNA) gene-linked short tandem repeats (STR) were amplified from the DNA using species-specific primers for *E. histolytica* and *E. dispar*. The primers used for the molecular characterization were DA-H5/H3 (Hsp 1–2), NK-H5/H3, S^tga^D-H5/H3, SQ5/SQ-H3 for *E. histolytica* and DA-D5/D3 (Dsp 1–2), NK-D5/D3, S^tga^D-D5/D3, SQ-D5/SQ-D3 for *E. dispar* [19].

These molecular markers amplify highly polymorphic intergenic sequences repeated in tandem (STR) associated to tRNA genes in both species of *Entamoeba*. In all cases, the PCR conditions were as follows: The reaction mixture consisted of 1 μL of clean extracted DNA added to Tris–HCl 10 mM, pH 8.3, KCl 50 mM, gelatin 0.001 %, MgCl_2_ 2 mM, 0.2 mM of each nucleotide, 0.0025 U of polymerase (AmpliTaq platinum Polymerase, Invitrogen) and 20 μM of each primer; 5 min at 95 °C for the initial incubation, followed by 35 cycles of 30 s at 95 °C, 30 s at 72 °C and a final extension step of 10 min at 72 °C. Regarding the primers, the annealing temperature conditions were those reported by Ali et al., 2005. The PCR products were subjected to electrophoresis in 1.5 % agarose gels stained with ethidium bromide and visualized in an UV transilluminator. Sequencing of PCR products was carried out in a reaction mixture (15 μL) consisting of 2 μL of the Big Dye Terminator Sequencing Kit (Applied Biosystems, San Francisco, CA), 1.6 μM of the appropriate primer, and 5 μL of the purified PCR product. The amplification conditions were as follows: 1 cycle of 5 min at 95 °C, 45 cycles of 10 s at 95 °C, 10 s at 50 °C, and 4 min at 60 °C. Sequencing was performed in a capillary sequencer (ABI-Avant 100, University of Washington). The sequences were manually verified using the BioEdit program [20]. Taxonomic identity was established by comparing the obtained sequences with those available in GenBank (NCBI). The sequences were aligned using the program CLUSTAL X [21]. Phylogenetic reconstruction based on the molecular marker NKD3-D5 was carried out through the Unweighted Pair Group Method with Arithmetic Mean (UPGMA) using the Statistical Package for the Social Sciences (SPSS), version 17.

### Genetic diversity

With NKD3-D5 sequences obtained from the school and family isolates, and those reported in GenBank, we conducted a genetic diversity analysis using the program DnaSP version 5.0 [22]. The number of both segregate sites and haplotypes was estimated, as well as the average of nucleotide diversity per site (π) and the expected variation, by assuming a neutral site evolution (θ).

### Estimation of the anemia index

The concentration of Hemoglobin (Hb) was determined from peripheral blood samples using a HemoCue analyzer (HemoCue AB, portable Hb Analyzer, Angelhom, Sweden). The values were stored in a database for analysis, contrasting the Hb g/dl with the standard curves of Hb concentration by age-group [23].

### Body Mass Index

Body weight is expressed as the mean of two independent measurements using electronic scales (Medical Scales and Measuring Systems, Seca, Hamburg, Germany). Height was measured with a portable stadiometer (Medical Scales and Measuring Systems, Seca), taking the mean of two measurements. The Body Mass Index (BMI) was calculated using the following formula: weight (kg)/[height (m)] ^2^. The BMI was used as an index of relative weight, with BMI-for-age *z* scores, and the percentiles were calculated according to age and sex using the Center for Disease Control and Prevention growth charts [24].

### Statistical analysis

The frequency of intestinal parasitic infections was estimated as the number of positive cases/total school population studied; to calculate the relative frequency of single or multiple parasitic infections, we used the same denominator.

The search for an association between sociodemographic variables, health and anthropometric values was done using Pearson’s chi-square and chi square for trend assessment. In all cases, the statistical significance was set at *p* less than 0.05.

## Results

### General characteristics of the studied cohort

The distribution of children by age and gender in each of the three studied schools was equal; the total number of participants was 309 children, 156 boys and 153 girls. Table 1 shows the sociodemographic variables in the family environment investigated as potential risk factors for parasitic diseases; these were: presence of harmful fauna (cockroaches, fleas, rats, mice, flies); piped water inside the household, type of latrine (water latrine or septic tank), floor material of household dwelling (cement or soil) and the quality of water used for human consumption. The differences in these variables between infected and non-infected children were not statistically significant (*p* > 0.05). Table 2 shows that the frequency of intestinal parasite infection was not associated with age or gender, with a similar distribution for males and females. With regard to the risk factors in the schools, a hygiene index was established based on the conditions of the sanitary facilities, the cleanliness of bathrooms, the availability of trash cans, the presence of clean common areas, overcrowding conditions and access to health authorities. Using this index, the schools were ranked as good, regular or bad, similar to Webb et al. description [25]. The results of the hygiene index and the frequency of parasitization among children (by school) were subjected to a chi-square test for linear trend analysis, which showed that the differences between the schools were not statistically significant (*p* = 0.74) (Table 3).

**Table 1:** Analysis of the association between sociodemographic variables at home and the parasitized or non-parasitized condition of school children

		Parasitized children				
Variables		Positive	Negative	^*a*^ *p*	OR	IC 95 %
Harmful fauna	Yes	65	218	0.73	1.63	(0.35–7.58)
	No	2	11			
Availability of water inside the house	Yes	65	224	0.27	0.53	(0.20–1.40)
	No	7	13			
Type of latrine	Latrine	67	214	0.26	2.66	(0.59–11.8)
	septic tank	2	17			
Floor material	Cement	58	210	0.12	0.52	(0.24–1.16)
	Soil	11	21			

^*a*^ Fisher’s exact test

**Table 2:** Children distribution by age, gender and parasitized or non-parasitized condition

		Parasitized children				
Age range (years)		Positive	Negative	^*a*^ *p*	OR	IC 95 %
5–9	Female	17	62	0.80	0.91	(0.44–1.89)
	Male	21	70			
10–14	Female	16	58	0.40	0.72	(0.33–1.56)
	Male	18	47			

^*a*^ Chi^2^ test

**Table 3:** Number of parasitized and non-parasitized children per school

School	Microscopic examination of stool samples			OR	IC 95 %
	Positive (%)	Negative (%)	Total		
Sofía Vázquez^a^	29 (23.2)	96 (76.8)	125	1	--
Lázaro Cárdenas^b^	13 (28.9)	32 (71.1)	45	1.34	(0.58–3.09)
Emiliano Zapata^c^	30 (21.6)	109 (78.4)	139	0.91	(0.49–1.69)
Total	72	237	309		

^a^Good hygiene index

^b^Regular hygiene index

^c^Bad hygiene index

Chi^2^-with lineal trend = 0.11 *p* = 0.74

Pathological antecedents specifically related to diarrhea episodes in the previous 12 months and the presence of mucus in the feces or blood were investigated in both parasitized and non-parasitized children; it was found that parasitized children had suffered 40 diarrhea episodes compared with 107 episodes among non-parasitized individuals; however, the differences between groups were not statistically significant (*p* = 0.11). With regard to bloody and mucous diarrhea, there were only eight such episodes in parasitized children.

### Microscopy frequency of intestinal parasitic infection

The frequency of intestinal parasitic infection in the studied population (*n* = 309 children) was 23.3 % (72/309). The frequency of single infections was (48/72) 66.6 %, and 24 individuals (33.3 %) showed multiple infections (more than one parasite species); some of these were intestinal pathogens. It is worth noting the high frequency of *Blastocystis hominis* (25 %), *Giardia lamblia* (19.4 %), and *E. histolytica*/*E. dispar* (9.7 %).

### Molecular frequency of *E. histolytica* and *E. dispar*

After microscopic screening for intestinal parasites, 72 stool samples were treated for DNA extraction and thereafter for PCR amplification; these were tested for *E. histolytica* and *E. dispar* using species specific primers; 30 out of 72 parasitized samples (41 %) were positive for *E. histolytica* and/or *E. dispar*; 8/72 samples corresponded to individuals infected with *E. histolytica* (11.1 %); 19/72 samples corresponded to *E. dispar* infected children (23.88 %); and 3 out of 72 samples belonged to children infected with both *E. histolytica* and *E. dispar* (4.16 %). With the purpose of establishing the dynamics of transmission between each child and their near relatives, we tested the relatives of the 72 parasitized stool samples and PCR tested for the presence of *E. histolytica* and *E. dispar*. There was a low frequency of infection in this group of studied individuals; only 14 out of 48 parasitized samples (29.2 %) were positive for *E. dispar*, and we did not detect infection with *E. histolytica* or mixed infections in this group (Tables 4, 5).

**Table 4:** *Entamoeba histolytica* and *Entamoeba dispar* frequency: microscopic and PCR analysis of stool samples of the school children

	PCR characterization				
Microscopy	*E. histolytica*	*E. dispar*	*E. histolytica* + *E. dispar*	Negative	Total
Eh/Ed	1	6	0	4	11
Non Eh/Ed	7	13	3	38	61
Negative	−	−	−	237	237
TOTAL	8	19	3	287	309

Values are the frequency of *E. histolytica* and/or *E. dispar* species microscopically and/or PCR detected. *E. histolytica* frequency [*E. histolytica* + (*E. histolytica* + *E. dispar*)] was 3.55 %; *E. dispar* frequency [*E. dispar* + (*E. dispar* + *E. histolytica*)] was 7.12 %, and frequency of *Entamoeba* infection [*E. histolytica* + (*E. histolytica* + *E. dispar*) + *E. dispar*] was 9.70 %. PCR, polymerase chain reaction. (−) PCR was not performed

**Table 5:** *Entamoeba histolytica* and *Entamoeba dispar* frequency: microscopic and PCR analysis of stool of the relatives of the parasitized school children

	PCR characterization				
Microscopy	*E. histolytica*	*E. dispar*	*E. histolytica* + *E. dispar*	Negative	Total
Eh/Ed	0	6	0	0	6
Non Eh/Ed	0	8	0	34	42
Negative	−	−	−	119	119
TOTAL	0	14	0	153	167

Values are the frequency of *E. histolytica* and/or *E. dispar* species microscopically and/or PCR detected. *E. histolytica* frequency [*E. histolytica* + (*E. histolytica* + *E. dispar*)] was 0′%; *E. dispar* frequency [*E. dispar* + (*E. dispar* + *E. histolytica*)] was 8.38, and frequency of *Entamoeba* infection [*E. histolytica* + (*E. histolytica* + *E. dispar*) + *E. dispar*] was 0 %. PCR, polymerase chain reaction. (−) PCR was not performed

### Sequence analysis based on the number of motifs

Although the PCR was performed using all primers mentioned above, the NKD3-D5 marker showed the best performance, and thus we decided to work with the PCR products obtained with this specific marker for *E. dispar*. The sequence profiles (GenBank accession number KX461938-KX461956) were analyzed, manually aligned and compared with those reported in GenBank.

**Fig. 1:**
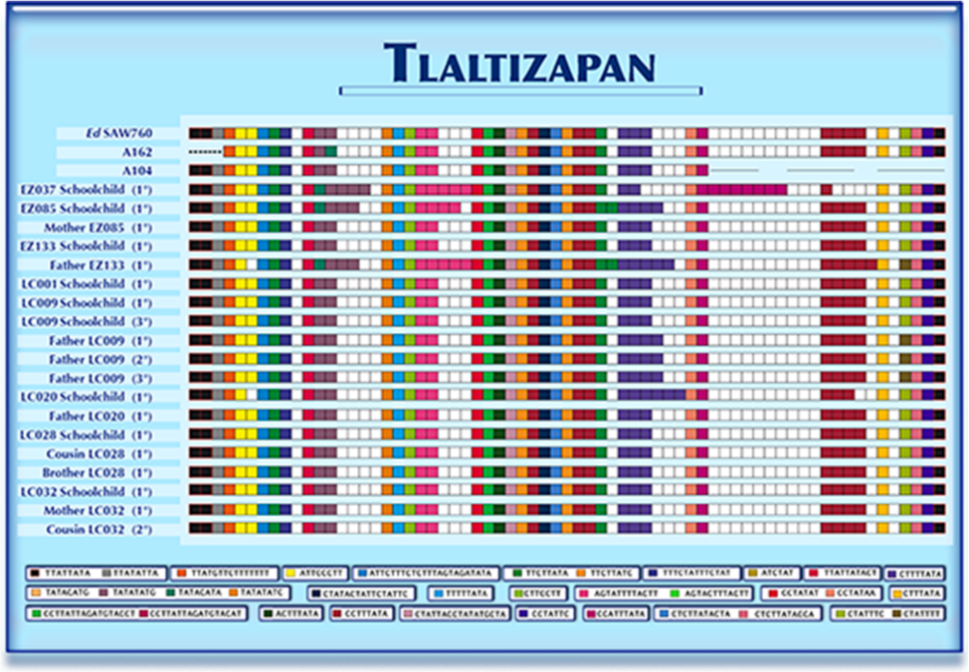
Schematic representation of the motifs found in isolates of schoolchildren, their families and sequences reported in GenBank with the NKD5-D3 marker of *E. dispar*

In order to establish the dynamics of infection of *E. dispar*, we analyzed sequences of school children and their families, including previous NKD3-D5 sequences reported in GenBank. The analysis of isolates using this molecular marker was based on the diversity and the number of motifs found in the sequences. Twenty-four different motifs were defined, each one represented with a specific color. In some cases, the motifs consisted of two or four sequences and we decided to include all of them into a single motif, since the differences between the sequences was only one nucleotide. Afterwards, we defined genetic patterns based on the number of repeated motifs; these patterns were compared between classmates and between the respective family members. The correlation study showed no clear route of transmission, since the different genetic patterns were shared between classmates and family members (Fig. 1).

### Genotypes

**Fig. 2:**
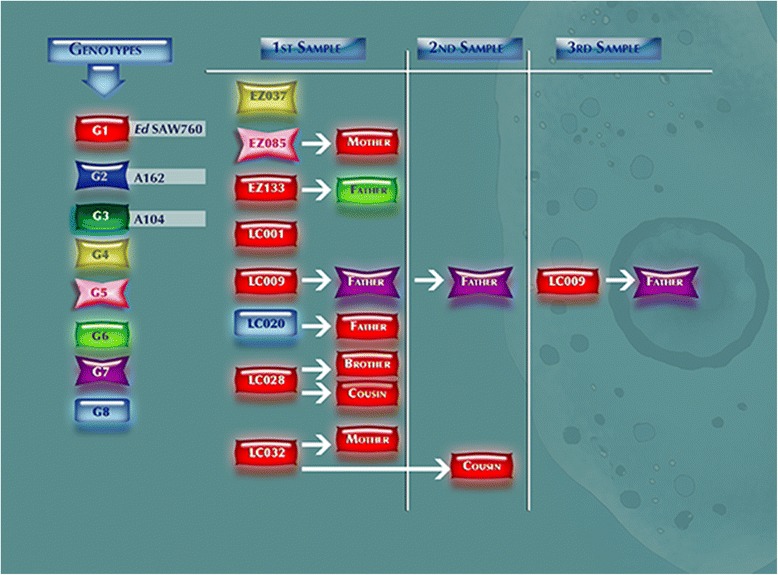
Representation of the relationship of the genotypes found in the schoolchildren and their immediate families at different sampling

Eight genotypes were found in parasitized children and their families; each genotype is represented by a color (Fig. 2). It appears that most of school children in Lazaro Cardenas school share genotype 1, the same genotype found in their closest relatives, except child LC020, whose genotype was genotype 8, entirely different to that of his classmates. Cases of persistent infection were also detected; for example, the child LC009, who also attended Lazaro Cardenas school, was infected by genotype 1 and his father was infected by *E. dispar* genotype 7; both remained infected throughout the entire follow-up. This phenomenon was not found in the Emiliano Zapata school, where there was more diversity of genotypes in the infected children, who did not share the genotypes with other classmates or with members of their respective families. The most frequent genotype was genotype 1, which, as we mentioned before, is the same showed by the reference strain SAW760 of *E. dispar* SAW760.

### Genetic diversity

Table 6 shows the genetic diversity of *E. dispar* detected in this community using the NKD3-D5 marker. In the group of 19 sequences obtained from children and their relatives, we found 6 haplotypes, considering insertions, deletions and substitutions of nucleotides; 8 segregative sites, and π and θ values of 0.00337 and 0.00622 respectively. When the analysis included all corresponding sequences available in GenBank, we found 8 haplotypes from 22 sequences, also considering insertions, deletions and substitutions, with 9 segregative sites, and π and θ values of 0.00147 and 0.00354 respectively.

**Table 6:** Parameters related to the genetic diversity of the sequences in the specie *Entamoeba dispar* using the molecular marker NKD3-D5 (603pb)

Groups^a^	No. of sequences	No. of haplotypes	Ss^b^	π	θ
School and Fam. *E. dispar*	19	6	8	0.00337	0.00622
*E. dispar* total	22	8	9	0.00114	0.00185

^a^School and Fam *E. dispar*: Number of samples of school children and their relatives obtained in this study; total *E. dispar* total: number of analyzed sequences available in the GenBank data

^b^Number of segregated sites

### Phylogenetic reconstruction

**Fig. 3:**
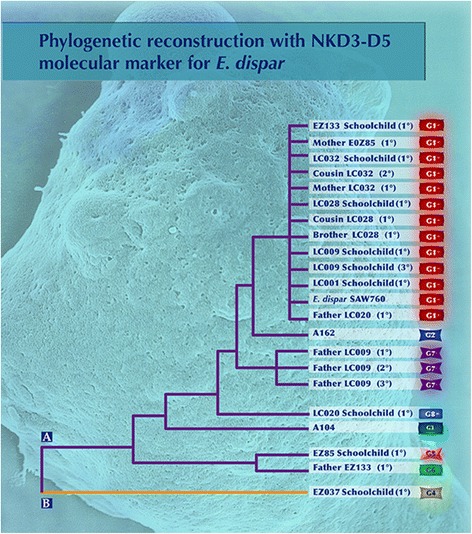
Phylogenetic representation of isolates obtained in schoolchildren, their families and sequences reported in GenBank

For the phylogenetic reconstruction of isolate sequences, we created a matrix where the number of motifs detected was encoded as present (1) or absent (0) with a numeric value. Thereafter, we used the Social Sciences (spss) statistical package version 17 to carry out the phylogenetic reconstruction using the Unweighted Pair Group Method with Arithmetic Mean (UPGMA). This analysis showed two major divergent groups, A and B. Fig. 3 shows that the majority of sequences from children and from their relatives belong to group A. As can be seen, this group is formed by subgroups; for example, subgroup I corresponds to sequences from Lazaro Cardenas school children and some of their relatives. These sequences are identical to the sequence of *E. dispar* SAW760 strain, which also belongs to group A, subgroup I. The rest of the sequences are dispersed among the other clades.

### Anemia index

We estimated the concentration of hemoglobin in peripheral blood samples (Hb g/dl) from 299 out of 309 children and compared it with reference data by age range for diagnostic criteria for anemia [23]; we found that 21 out of 299 children had Hb g/dl values that, for their age, were indicative of anemia (6.9 %). However, the differences in Hb g/dl levels between the groups of parasitized and non-parasitized children were not statistically significant (*p* = 0.53) (Table 7).

**Table 7:** Analysis of the association between hemoglobin and intestinal parasitic infection

	Microscopic examination of stool samples			
Hemoglobin Levels	Positive (*n* = 67)		Negative (*n* = 232)	
		%		%
Hb^a^	5	7.46	16	6.89
Hb^b^	62	92.5	216	93.1

^a^ children with Hb levels associated to anemia (Lanzkowsky [23])

^b^ children with normal levels of Hb

Exact Fisher’s test *p* = 0.79

### Anthropometric characteristics of the study population

The body mass index (BMI) calculations by sex and age of the 309 studied children showed differences in the population according to sex and age (data not shown). Over 50 % (192) of the children were between the 5th and 85th percentile, which corresponds to normal BMI. However, 13 children (4.2 %) were under the 5th percentile, which is considered underweight; 57 children (18.6 %) had a BMI between the 85th to 95th percentile, which defined overweight; moreover, 44 (14.3 %) children were classified as obese (over the 95th percentile). When the BMI was correlated with intestinal parasitic infection, the differences detected were not statistically significant (*p* = 0.41) (Table 8).

**Table 8:** Anthropometric characteristics: association between body mass index (BMI) and parasitized status

Microscopic examination (*n* = 306)	<5th percentile (under-weight)	5th to 85th percentile (normal weight)	85th to 95th percentile (over-weight)	≥95th percentile (obese)	Total (%)
Parasitized	3	51	10	8	72 (23.5)
Non-parasitized	10	141	47	36	234 (76.4)

Chi^2^ test = 2.85, *p* = 0.41

## Discussion

Intestinal infections due to intestinal pathogens are endemic in developing countries all over the world [26]. Intestinal parasitic infections are closely associated with specific sociodemographic risk factors in the community environment, and particularly at home (Table 1). In addition, the highest rates of parasite infection were found in children aged 1–4 years (Table 2); however, no differences were found in the sociodemographic variables studied between infected and non-infected children. Furthermore, differences in age or gender between parasitized and non-parasitized children were not statistically significant (*p* >0.05. This suggests that the main sources of infection for the children may be outside the family environment. The other place where children spend most of their time is the school. The three schools showed differences in the hygiene index, and we expected to find some important differences in the parasitized condition of children; however, the differences between the hygiene index of schools were not statistically significant (chi-square for linear trend = 0.11; *p* = 0.74). In summary, the demographic variables of family, age, gender and school environment of the studied children were not directly responsible for the intestinal parasitic infection (Tables 1, 2 and 3); this circumstance points out to the existence of wide spread sources of intestinal parasites in the community environment.

Actually, the frequency of intestinal parasitic infection in the state of Morelos (23.3 %) is not the highest in our country compared with those observed in rural communities in the neighboring states of Guerrero or Chiapas, where the recently reported prevalence of intestinal parasitic infection exceeds that observed in Tlaltizapan [26, 27].

Our results on the frequency of pathogenic parasites in 2011 in Tlaltizapan can be contrasted with those obtained in 2005 in a rural community located 34 km southeast of Tlaltizapan [5]. In both cases, we detected protozoan pathogens such as *E. histolytica*, *E. dispar* and *G. lamblia*, in addition to a number of protozoa indicators of fecalism. However, the changes in the frequency of intestinal protozoan infection in children are worth noting; the frequency of *Blastocystis sp* in our children was remarkably high (25 %), in contrast to the complete absence of *Blastocystis sp*. observed in our previous study [5], although this figure is similar to that observed in other countries such as Thailand, Philippines or Brazil, where the prevalence are 20–40 % in 5–10 years old children [28, 29].

Intestinal parasites are etiological agents of diarrhea in both adults and children; however, children under 15 years old are the main group at risk. Children under 5 years old develop serious adverse effects related to cognitive skill, nutritional status and short stature, as consequence of repeated diarrhea events [30]. In our cohorts, we documented cases of diarrhea in both parasitized (55.5 %) and non-parasitized (44.9 %) children; however, the differences between the groups were not statistically significant. Clinically, none of the cases of diarrhea was attributed to an amoebic disease; in all cases the outcome was treated with oral rehydration solution. Some diarrheal events were self-limiting and resolved spontaneously within 48–72 h; in some other cases, the physician in charge indicated oral antibiotic administration.

It is worth noting the evident tolerance of the school children to intestinal parasitic infection, not only to those parasites considered as commensal but also to pathogenic parasites. Furthermore, it appears that parasitism has no impact on the anemia index (Table 7); actually, the overall frequency anemia was 6.9 %, but the differences between infected and non-infected groups were not statistically significant. This contrasts with previous studies that reported the presence of iron deficiency anemia in children with repetitive events of diarrhea [31, 32].

Furthermore, intestinal parasitic infection did not substantially affect the growth and development of the children, as indicated by the analysis of differences in the BMI between infected and non-infected groups (*p* < 0.05) (Table 8); nevertheless, there are studies in which the presence of parasitism had detrimental effects on the growth and development of children with repeated episodes of diarrhea [31, 32].

We should look for cryptosporidium and coccidian infection in the feces samples of the children studied here; however, we decided not to use the kinyoun staining method due to the irrelevant frequency of these parasites in the state of Morelos compared with the frequency of these infections in other states of Mexico [33].

The availability of molecular tools to study the molecular epidemiology of *E. histolytica* and *E. dispar* infection in endemic regions allows us to determine the distribution of genetic variants of the two species in different geographic regions; hypothetically, knowing the geographic distribution of virulent genotypes of *E. histolytica* may help predict possible morbidity surges in at risk communities. Unfortunately, hitherto there is no convincing evidence of the relationship between genotypes and the type of amoebic outcome of the infected host. Efforts in this direction have been made using a number of polymorphic molecular targets [15, 34, 35]. The most studied molecular targets used to analyze the association of genotypes with the different outcomes of disease (amebic colitis, amebic liver abscess or asymptomatic intestinal infection) are the STRs of the intergenic region related to tRNA genes [19, 36]. These were the targets selected for our study; the NKD3-D5 marker was the one that produced the best PCR products and sequences in a large number of samples. The NKD3-D5 marker allowed us to indentify 8 different genotypes in the studied children and their relatives; the most prevalent was genotype 1 (Fig. 2).

It is worth noting that the Lazaro Cardenas school, which has particular characteristics such as overcrowded classrooms, was the school attended by most of the school children belonging to a close community in Tlaltizapan; the people of this community live in extreme poverty. This school was the one with the lowest value in the hygiene index (Table 3); it was also the school with the highest frequency of intestinal parasitic infection (28.9 %). Given these facts, one would expect a higher frequency of *E. histolytica*, and greater genetic diversity of *E. histolytica*. However, it seems to be a common phenomenon, which has been observed in other endemic communities, that the same genotypes circulate from one individual to another within the community; in our study, the transmission patterns of the parasites went from the homes to the school and viceversa [5, 12, 13, 37, 38].

The analysis of the genetic diversity of *E. dispar* isolates obtained in our group of children and their relatives using the NKD3-D5 marker showed what can be considered a low diversity (Table 6), since we obtained only 6 genotypes from 19 sequences with 8 segregative sites (Ss), π value of 0.00337 and θ of 0.00622. Considering the total number of sequences (our sequences and those available in the databases), the values were: 8 genotypes from 22 sequences, 9 Ss, π and θ values of 0.00114 and 0.00185 respectively; these values correspond to a low diversity.

Previously, we reported the genetic diversity of *E. dispar* isolate from amoebic liver abscess material in which, using the D-A marker, we obtained 15 genotypes and π and θ values of 0.081 and 0.064 respectively. Even though there is not enough information about the genetic diversity estimated through the π and θ rates, the previous values are higher than those obtained in the present work [14]. Escueta-de Cadiz et al. (2010) [39] reported the genotyping of *E. histolytica* using the marker NK2H3-H5; they identified 5 motifs with 8 nucleotides each, which indicates that *E. histolytica* is less polymorphic than *E. dispar*; the use of the D-A genetic marker [13] demonstrated that *E. histolytica* is indeed less polymorphic than *E. dispar*. The low genetic diversity of our isolates suggests that most of the segregative sites are found in only a few sequences.

## Conclusions

The comparison of the genotypes of *Entamoeba* species did not show correlation between children and their relatives; this was also observed in the most frequent genotypes in the schools, suggesting that in this community the species of *E. dispar* genotype 1 is the most widespread.

Finally, based on the indicators of growth, development and nutrition status, we can conclude that the studied community seems to be reasonably adapted to constant exposition to intestinal parasites, not only to those considered commensal but also to pathogens, since there were no evidences of a serious impact of the parasitized condition on the children’s health.

## Abbreviations

BMI: Body mass index
Hb: Hemoglobin
PCR: Polymerase chain reaction
Ss: Segregative sites
STR: Short tandem repeats
UPGMA: Unweighted pair group method with arithmetic mean
